# Human Breast Milk‐Derived Exosomal FP671120.4 Inhibits Macrophage M1 Polarization via Modulating the ELAVL1/Nrf2 Axis in Sepsis‐Associated Liver Injury

**DOI:** 10.1002/kjm2.70108

**Published:** 2025-09-27

**Authors:** Zhao‐Bin Yang, Yi‐Bin Gao, Xiao‐Mei Cheng, Lu‐Zhen Qiu

**Affiliations:** ^1^ Department of Internal Medicine ICU Zhangzhou Hospital Affiliated to Fujian Medical University, Zhangzhou Municipal Hospital of Fujian Province Zhangzhou China

**Keywords:** ELAVL1, FP671120.4, HBM‐exos, M1 polarization, Nrf2/HO‐1

## Abstract

Sepsis‐associated liver injury (SALI) plays a major role in aggravating disease progression and worsening prognosis in patients with sepsis. Macrophage polarization is a key factor in the modulation of SALI progression. Recent studies have shown that human breast milk‐derived exosomes (HBM‐Exos) regulate processes involved in macrophage polarization. Here, we investigated the function and mechanism of action of HBM‐Exos in a macrophage polarization model of SALI. The extracted HBM‐Exos were identified by morphological analysis and detection of marker proteins using flow cytometry. Human Kupffer cells were treated with lipopolysaccharide (LPS) to simulate macrophage polarization in SALI. Cell viability was measured using a CCK‐8 kit. Protein and gene expression levels were evaluated using western blotting and RT‐qPCR, respectively. ELISA kits were used to assess the levels of inflammatory cytokines. The interactions between FP671120.4, ELAV Like RNA binding protein 1 (ELAVL1), and nuclear factor erythroid 2‐related factor 2 (Nrf2) were verified by RIP analysis. HBM‐Exos inhibited M1 macrophage polarization by promoting Nrf2 expression and phosphorylation via activation of the Nrf2/Heme oxygenase‐1 (HO‐1) signaling pathway in LPS‐induced Kupffer cells. Furthermore, FP671120.4 reversed the HBM‐Exos‐mediated increase in Nrf2 mRNA stability. HBM‐Exos‐derived FP671120.4 enhanced the interaction between ELAVL1 and Nrf2. As a result, FP671120.4 inhibited M1 polarization by inducing Nrf2 expression via activation of the Nrf2/HO‐1 pathway. These findings suggest that HBM‐Exos‐derived FP671120.4 may inhibit M1 macrophage polarization through the ELVAL1/Nrf2/HO‐1 signaling pathway in LPS‐induced Kupffer cells.

**Abbreviations**
BP: Base pairs
CCK‐8: Cell counting kit 8
DMEM: Dulbecco's Modified Eagle Medium
ELAVL1: ELAV Like RNA Binding Protein 1
ELISA: Enzyme‐linked immunosorbent assay
FBS: Fetal bovine serum
GO: Gene Ontology
HBM: Human breast milk
HBM‐Exos: Human breast milk‐derived exosome
HO‐1: Heme oxygenase‐1
IL‐10: Interleukin‐10
IL‐6: Interleukin‐6
KEGG: Term and Kyoto Encyclopedia of Genes and Genomes
LncRNAs: Long non‐coding RNAs
LPS: Lipopolysaccharide
Nrf2: Nuclear factor erythroid 2‐related factor 2
NTA: nanoparticle tracking analysis system
PBS: Phosphate buffer saline
RIP: RNA immunoprecipitation
RT‐qPCR: Real‐time quantitative polymerase chain reaction
SALI: Sepsis‐associated liver injury
TEM: Transmission electron microscope
TNF‐α: Tumor necrosis factor‐α

## 1 Introduction

Sepsis is defined as life‐threatening organ dysfunction caused by a dysregulated host response to infection [1]. Uncontrolled inflammatory reactions induce significant dysfunction or injury to cells and organs, resulting in a high mortality rate associated with multiple organ dysfunction syndrome [2]. Sepsis‐associated liver injury (SALI) is a major predictor of mortality in patients with sepsis (approximately 62% of cases) and negatively impacts disease prognosis [3]. Current management strategies for SALI primarily address the underlying infection, but they are limited by side effects, suboptimal efficacy, and lack of specificity [4]. Therefore, there is an urgent need for a deeper understanding of the pathogenic mechanisms and related therapeutic targets to better control SALI progression.

Liver macrophages play a central role in the occurrence and development of SALI and are a primary component of the host immune defense [5]. Regulation of macrophage polarization, including the inhibition of pro‐inflammatory M1 polarization and promotion of anti‐inflammatory M2 polarization, has been shown to potentially alleviate SALI [6]. Kupffer cells, the liver's resident macrophages, drive SALI progression through M1 polarization and the excessive release of pro‐inflammatory cytokines [7]. Nuclear factor erythroid 2‐related factor 2 (Nrf2) is a key regulator of hepatic injury protection during SALI, and its upregulation is associated with disease alleviation in SALI mouse models [8]. The Nrf2/heme oxygenase‐1 (HO‐1) signaling pathway plays a vital role in inhibiting M1 polarization and markedly reduces inflammatory injury related to SALI [9]. However, the upstream mechanisms of macrophage polarization and Nrf2/HO‐1 signaling in SALI remain poorly understood and require further investigation.

Human breast milk (HBM) is a vital source of nutrients for infant growth and development and plays a crucial role in immune system modulation [10]. Numerous studies have shown that HBM enhances host defense responses in infants [11]. Moreover, HBM can exert protective effects against inflammation‐associated tissue injuries, such as necrotizing enterocolitis and chronic apical periodontitis [12, 13]. HBM has also been implicated in reducing the risk of sepsis in infants [14]. HBM‐derived vehicles, including exosomes, microbiomes, and oligosaccharides, play significant roles in the regulation of inflammation [15]. Among these, HBM‐derived exosomes (HBM‐Exos) have been shown to contribute to the regulation of inflammatory homeostasis in various diseases [16]. HBM‐Exos can modulate macrophage activity and influence inflammation by delivering functional RNAs directly into macrophages [17]. However, the precise role and mechanism of action of HBM‐Exos in SALI, particularly in the regulation of macrophage polarization, remains unclear and requires further investigation.

Long noncoding RNAs (lncRNAs) are RNA molecules that exceed 200 nucleotides in length and do not code for proteins but function as regulatory elements. Exosome‐derived lncRNAs exert crucial modulatory effects on cellular processes during disease progression. For instance, the sepsis‐associated encephalopathy (SAE) mouse serum‐derived exosomal lncRNA NEAT1 was found to exacerbate SAE by enhancing ferroptosis [18]. Notably, HBM‐derived exosomal lncRNAs have been shown to exert anti‐inflammatory effects in intestinal inflammatory diseases such as necrotizing enterocolitis [19]. However, their roles in the progression of other diseases, including SALI, remain largely unexplored, warranting further research.

In this study, we found that HBM‐Exos inhibited M1 polarization in lipopolysaccharide (LPS)‐treated Kupffer cells. Moreover, we identified a novel HBM‐Exo‐derived lncRNA, FP671120.4, and confirmed its ability to upregulate Nrf2 mRNA stability by recruiting ELAV‐like RNA‐binding protein 1 (ELAVL1), thereby inhibiting M1 polarization in LPS‐induced Kupffer cells. This investigation into the specific role and mechanism of HBM‐Exos may support their therapeutic potential in the treatment of SALI. Additionally, the ELAVL1/Nrf2 axis may serve as a promising molecular target for the early diagnosis and intervention of SALI.

## 2 Materials and Methods

### 2.1 Extraction and Identification of Human Breast Milk‐Derived Exosomes

HBM samples were acquired from the milk bank of the Affiliated Zhangzhou Hospital of Fujian Medical University, with collection conducted under the approval of the hospital's Medical Ethics Committee [No. 2023kyz281]. Exosomes from the HBM were extracted using density gradient centrifugation according to the corresponding protocol [20]. Briefly, HBM samples were centrifuged at 2000×g for 30 min (4°C), followed by 10,000×g for 45 min (4°C). The resulting supernatant was filtered through a 0.45 μm filter to discard the upper layer. The filtrate was then centrifuged at 100,000×g for 70 min to obtain the exosome‐containing pellet, which was resuspended in phosphate‐buffered saline (PBS). The exosome solution was supplemented with a 30% sucrose cushion (D2O) and centrifuged at 100,000×g for 70 min. Subsequently, 250 μL of the 30% sucrose cushion from the lower layer was removed, diluted with PBS, and then centrifuged again at 100,000×g for 70 min. The supernatant was discarded. To identify HBM‐extracted exosomes (HBM‐Exos), transmission electron microscopy (TEM, 1200EX II, JEOL, Beijing, China) was used to observe exosome morphology. A nanoparticle tracking analysis system (NTA, ZetaView, Particle Metrix, Meerbusch, Germany) was used to analyze the exosomal particle size and distribution, following the detailed guidelines [20]. Classic exosomal marker proteins, including CD9 (#555371, BD Biosciences, Shanghai, China) and CD81 (#551108, BD Biosciences), were validated by flow cytometry analysis, as described in a previous study [21].

### 2.2 Cell Culture and Treatment

Human Kupffer cells were obtained from Otwobiotech (#HTX1973, Shenzhen, China) and cultured in Dulbecco's Modified Eagle Medium (DMEM, Gibco, Shanghai, China) supplemented with 10% fetal bovine serum (FBS, Gibco) and 1% antibiotics. Cells were maintained in a humidified incubator at 37°C and 5% CO_2_ under standard conditions. To establish a cell model of sepsis‐associated liver injury, Kupffer cells were stimulated with lipopolysaccharide (LPS) at a concentration of 1 μg/mL for 16 h, as described in previous studies [2]. The morphology of Kupffer cells, with or without LPS stimulation, was observed under a microscope (CX43; Olympus Corporation, Shanghai, China). Moreover, Kupffer cells were treated with LPS and HBM‐Exos (5, 10, and 15 μg/mL) to investigate potential interaction mechanisms.

### 2.3 Cell Counting Kit 8 (CCK‐8) Assay

Kupffer cell viability was determined using a CCK‐8 kit (#CA1210, Solarbio Biotech, Beijing, China) according to the manufacturer's instructions. In brief, Kupffer cells (1 × 10^3^ cells/well) were seeded into a 96‐well plate and incubated for 1 h at 37°C. Subsequently, CCK‐8 solution (10 μL) was added to each well, followed by another 3 h incubation. The optical density value (450 nm) was measured using a Spark microplate reader (Tecan Laboratory Equipment, Shanghai, China).

### 2.4 Enzyme‐Linked Immunosorbent Assay (ELISA)

Proinflammatory cytokines in the supernatant of Kupffer cells, including tumor necrosis factor‐α (TNF‐α), interleukin‐6 (IL‐6), and interleukin‐10 (IL‐10), were quantified using ELISA kits, according to the manufacturer's instructions (Elabscience Biotech, Wuhan, China). Absorbance was measured using a microplate reader (Tecan Laboratory Equipment, Shanghai, China).

### 2.5 Lentivirus Vector Infection

To knock down Nrf2, FP671120.4, and ELAVL1 in Kupffer cells, the corresponding coding sequences were designed and subcloned into the lenti‐vector plasmid HBLV‐U6‐MCS‐PGK‐Puromycin (Hanbio Biotech, Shanghai, China). The recombinant lentiviruses were packaged by co‐transfecting the lenti‐vector plasmid and packaging plasmids (Hanbio Biotech) into 293T cells. Kupffer cells (1 × 10^5^ cells/well) were infected with the harvested recombinant lentiviruses in culture medium supplemented with polybrene (3 μg/mL) for 24 h. After infection, cells were maintained in fresh Kupffer cell culture medium. Finally, protein and mRNA expression levels were assessed to confirm transduction efficacy.

### 2.6 Flow Cytometry Analysis

To evaluate M1 polarization in Kupffer cells, classical markers, including CD86 and F4/80, were analyzed by flow cytometry, following the protocol described in a previous study [22]. Briefly, Kupffer cells (1 × 10^6^ cells/well) were incubated with CD86 (10 μL, #305411, BioLegend, San Diego, CA, USA) and F4/80 (10 μL, #123107, BioLegend) antibodies for 30 min at 4°C. After washing with PBS, the proportion of CD86^+^F4/80^+^ cells was determined using a flow cytometer (CytoFLEX Nano, Beckman Coulter) and analyzed using FlowJo software.

### 2.7 Real‐Time Quantitative Polymerase Chain Reaction (RT‐qPCR)

Total cellular and exosomal RNA were isolated using the MolPure TRIeasy Plus Total RNA Kit (#19211ES60, Yeasen Biotech) and an Exosomal RNA isolation kit (#abs60263, Absin Biotech, Shanghai, China). cDNA conversion and qPCR were conducted using a One‐Step RT‐qPCR Kit (SYBR Green, #FP313; Tiangen Biotech, Beijing, China) on a Roche LightCycler 96 system (Roche, Basel, Switzerland). GAPDH was used as the internal reference gene. Relative mRNA levels of target genes were calculated using the 2^−ΔΔCt^ method. The primer sequences used and the accession numbers for each gene are listed in Table S1 of the Supporting Information.

### 2.8 Western Blot Analysis

Total cellular protein was isolated using a Nuclear and Cytoplasmic Protein Extraction Kit (#20126ES60, Yeasen Biotech). A BCA Protein Assay Kit (#P0012, Beyotime Biotech, Shanghai, China) was used to evaluate protein concentration. Protein samples (15 μg) were separated by electrophoresis on 15% SDS‐PAGE gels and transferred to polyvinylidene fluoride membranes (Absin Biotech). Membranes were blocked with 5% bovine serum albumin solution for 1 h (25°C) and incubated with the anti‐Nrf2 (1:1000, #ab62352, Abcam, Cambridge, UK), anti‐p‐Nrf2 (phospho S40, 1:1000, #ab76026, Abcam), and anti‐HO‐1 (1:1000, #ab189491, Abcam) primary antibodies overnight at 4°C. Subsequently, the relevant secondary antibodies (HRP‐labeled) were used to incubate the bands for an additional 1 h at 25°C. Finally, protein signals were visualized using West Pico ECL Substrate (#PE0020; Solarbio Biotech, Beijing, China) on an ImageQuant LAS 4000 system (GE Healthcare, Piscataway, NJ, USA). Histone H3 (1:500, #ab1791, Abcam) and β‐actin (1:500, #ab8226, Abcam) were used as normalization control proteins.

### 2.9 HBM‐Exos lncRNA Sequencing

lncRNA sequencing of HBM‐Exos was performed by Lifeint Biotech (Xiamen, China). Exosomal RNA was isolated using an Exosomal RNA isolation kit (Absin Biotech), according to the manufacturer's instructions. Isolated RNA was treated with DNase I (#D2215, Thermo Fisher Scientific) to remove genomic DNA contamination. RNA samples (4 μL) were then subjected to microamplification (13 cycles), using the RNALib Single Cell WTA Kit (#A5001, Lifeint) and following the manufacturer's instructions. After purification with magnetic beads, cDNA was obtained, and its concentration was measured using the QuantiFluor dsDNA System (#E2670, Promega, Beijing, China). For library preparation, 1 ng of cDNA was used following the instructions of the Lifeint Transpose DNA Library Prep Kit for Illumina (#A5006, Lifeint). Thirteen amplification cycles were carried out, and the final library was obtained after magnetic bead purification. The concentration of the resulting library was determined using the QuantiFluor dsDNA System (Promega), and the fragment size was evaluated using the Qsep100 S2 system. Before size evaluation, the library was diluted using Dilution Buffer (1 μL) to achieve an appropriate concentration. Sequencing was performed in PE150 mode on the Illumina NovaSeq 6000 high‐throughput platform. Differential expression was assessed using DESeq with read counts as input. Multiple Benjamini–Hochberg corrections were performed. Differentially expressed genes were selected based on a fold change > 2 and an adjusted *P*‐value < 0.05. The raw data were deposited in the Genome Sequence Archive of the China Bioinformatics Center.

### 2.10 Identification of New lncRNAs

Transcripts with uncharacterized functions were analyzed to identify potential long non‐coding RNAs (lncRNAs). Thresholds for the minimum length and exon count were applied to differentiate potential protein‐coding RNAs from non‐coding RNAs. Transcripts with lengths greater than 200 nucleotides and predicted open reading frames of less than 300 nucleotides were retained as candidate lncRNAs. To further refine the selection and classification of these candidates, computational tools including CPC, CNCI, and Pfam were employed. The top ten enriched lncRNAs were subsequently validated by RT‐qPCR analysis.

### 2.11 Differential Expression Analysis

Differentially expressed genes were identified using DESeq, DESeq2, edgeR, or DEGseq software, based on an adjusted *P*‐value < 0.05 and an absolute log_2_ (fold change) > 1.5. Hierarchical clustering analysis was then conducted using the “gplots” package in *R*. Clustering was based on the transcripts per million values of differentially expressed genes between the different groups. Color was used to display different clustering information, illustrate similarities in expression patterns within the same group, and indicate common biological functions or involvement in shared biological processes.

### 2.12 Functional Enrichment Analysis

Gene Ontology (GO)‐Term and Kyoto Encyclopedia of Genes and Genomes (KEGG) pathway enrichment analyses were performed to identify biological processes and pathways associated with differentially expressed genes. These analyses were performed using the clusterProfiler package in the R Bioconductor framework, with additional screening through the lncTarD database (http://bio‐bigdata.hrbmu.edu.cn/LncTarD/). The clusterProfiler package was used to identify and visually represent the enriched Gene Ontology (GO) terms and Kyoto Encyclopedia of Genes and Genomes (KEGG) pathways associated with all variants.

### 2.13 HBM‐Exos‐Derived FP671120.4 Stability Evaluation

Fresh HBM‐Exos were extracted from different HBM donors (*n* = 20). The expression levels of exosomal protein markers CD63 (#ab134045, Abcam) and TSG101 (#ab125011, Abcam) were determined by western blot analysis, while the levels of FP671120.4 were quantified using RT‐qPCR. The coefficient of variation (CV) in FP671120.4 expression across donors was calculated to evaluate its biological stability. To assess the stability of FP671120.4 RNA and HBM‐Exos under various conditions, HBM‐Exos were stored at −80°C, 4°C, and room temperature, and analyzed at multiple time points (0, 24, 72 h, and 1 week). RNA integrity was assessed via RT‐qPCR, and exosomal structure was examined using transmission electron microscopy (TEM).

### 2.14 RNA Immunoprecipitation (RIP) Assay

RIP analysis was performed to validate the interactions between FP671120.4, ELAVL1, and Nrf2, using an RNA Binding Protein Immunoprecipitation (RIP) kit (#B605109, Sangon Biotech, Shanghai, China). Kupffer cell lysates (containing intact RNA‐protein complexes) (10 μg) was incubated with protein A/G magnetic beads coated with anti‐ELAVL1 (4 μg, #ab200342) and anti‐IgG (4 μg, #ab172730) antibodies from Abcam for 1 h at 4°C. Subsequently, the coprecipitated RNA was isolated using protease K (#A414170, Sangon Biotech). The mRNA expression of Nrf2 and PF671120.4 was evaluated by RT‐qPCR analysis, while the efficiency of ELAVL1 immunoprecipitation was validated by western blotting.

### 2.15 RNA Decay Assay

Kupffer cells were treated with Actinomycin D (5 μg/mL, #M4881, Abmole Bioscience, Houston, TX, USA) for 0, 2, 4, 6, and 8 h. Then, Kupffer cells were harvested, and Nrf2 mRNA expression levels were assessed by RT‐qPCR.

### 2.16 Statistical Analysis

Statistical analyses were performed using SPSS software (version 29.0; SPSS Inc., Chicago, IL, USA). For comparisons between two groups, Student's t‐test was used. For multiple group comparisons, one‐way analysis of variance (ANOVA) followed by Tukey's post hoc test was applied. Pearson's correlation analysis was used to validate the relationship between the expression of FP671120.4 and Nrf2. Data are presented as mean ± standard deviation. Each experiment was independently repeated at least three times. A *P < 0.05* was considered statistically significant.

## 3 Results

### 3.1 Isolation and Identification of HBM‐Exos

**FIGURE 1:**
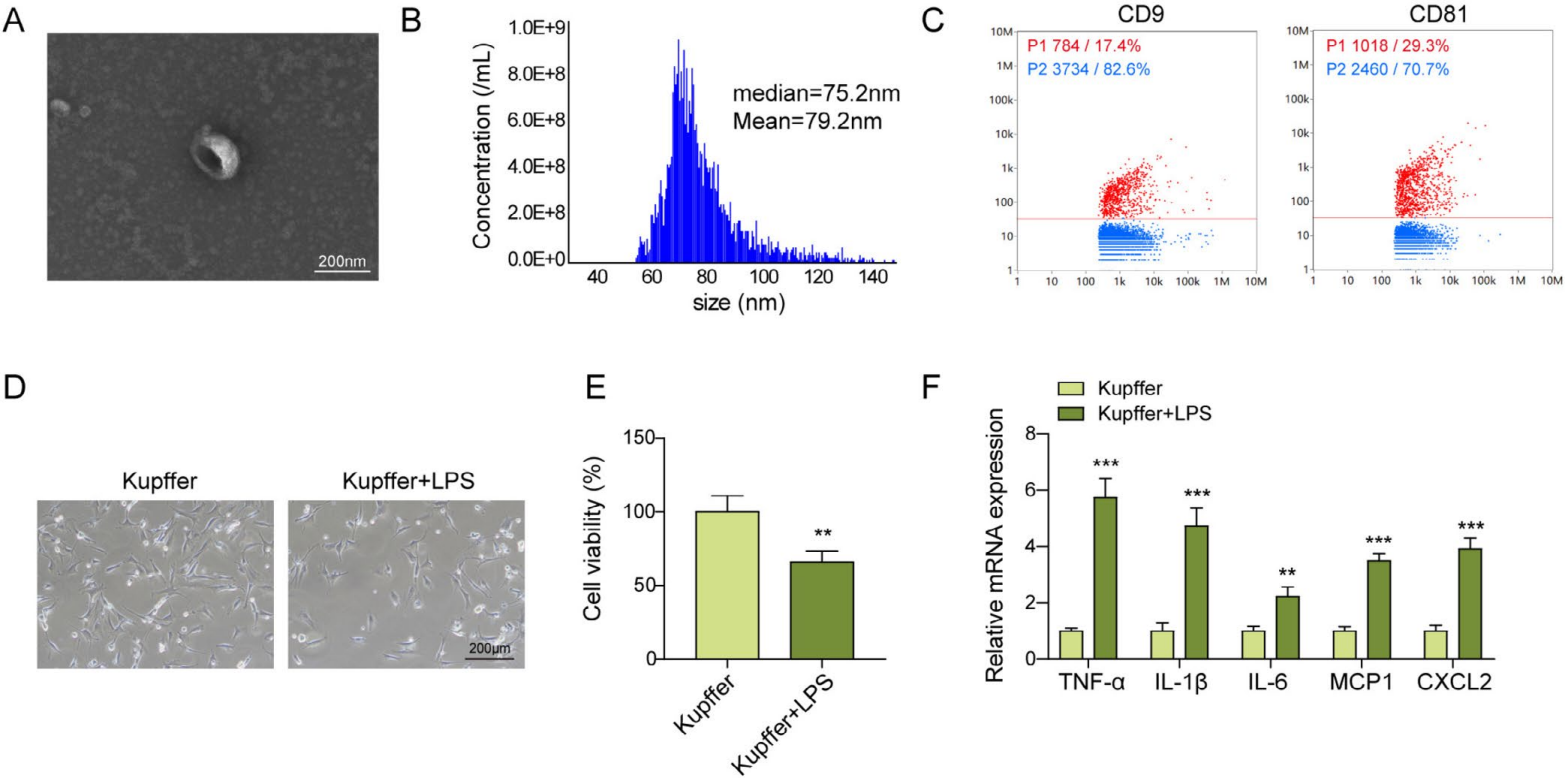
Isolation and identification of HBM‐Exos. (A) A transmission electron microscope was used to observe exosomal morphologies. (B) The exosomal particle size was determined using a nanoparticle tracking analysis system. (C) Flow cytometry was used to detect exosomal markers CD9 and CD81. Kupffer cells were stimulated with LPS (1 μg/mL) for 16 h (D–F). (D) An optical microscope was utilized to monitor cellular morphologies. (E) Cell viability was evaluated using the CCK‐8 assay. (F) RT‐qPCR analysis was used to detect the levels of TNF‐α, IL‐1β, IL‐6, MCP‐1, and CXCL2 cytokines. Each detection was performed in triplicate. Data are presented as means ± SD, *n* = 3 per group. **p < 0.01; ***p < 0.001.

To clarify the interaction between HBM‐Exos and LPS‐induced M1 polarization in Kupffer cells, HBM‐Exos were extracted from human breast milk following a previously established protocol [20]. Morphologically, HBM‐Exos were characterized by a double membrane and cup‐shaped vesicle, with a size distribution of approximately 30 to 1000 nm (median = 75.2 nm; mean = 79.2 nm) (Figure 1A,B). Furthermore, exosomal markers, including CD9 and CD81, were significantly enriched in the isolated exosomes, indicating successful extraction of HBM‐Exos (Figure 1C). To model hepatic macrophage M1 polarization, Kupffer cells were treated with LPS (1 μg/mL) for 16 h, as previously described [23]. Compared with the control group, LPS treatment significantly reduced the size and viability of Kupffer cells (Figure 1D,E). Additionally, LPS markedly upregulated the mRNA levels of inflammatory cytokines in Kupffer cells (Figure 1F). Although some inter‐donor variation in FP671120.4 expression was observed among exosomes from 20 individual donors, the coefficient of variation was within an acceptable limit, registering a value of 13.41% (< 15%) (Figure S1A). Furthermore, the expression levels of exosomal markers CD63 and TSG101 also remained stable across different samples, suggesting minimal variability in exosome yield (Figure S1B). HBM‐Exos were kept at −80°C, 4°C, and ambient temperature, and analyzed at various time intervals (0, 24, 72 h, and 1 week). The RNA integrity of FP671120.4 was maintained, as indicated by distinct electrophoretic bands. Further, qPCR analysis revealed no significant alterations in expression levels when compared to the baseline at −80°C (Figure S2A,B). Furthermore, electron microscopy revealed no morphological alterations in Exos stored at −80°C (Figure S2C). Collectively, these results confirm the successful isolation and stable characterization of HBM‐derived exosomes.

### 3.2 HBM‐Exos Inhibit Macrophage M1 Polarization and Activate Nrf2/HO‐1 Signaling in LPS‐Stimulated Kupffer Cells

**FIGURE 2:**
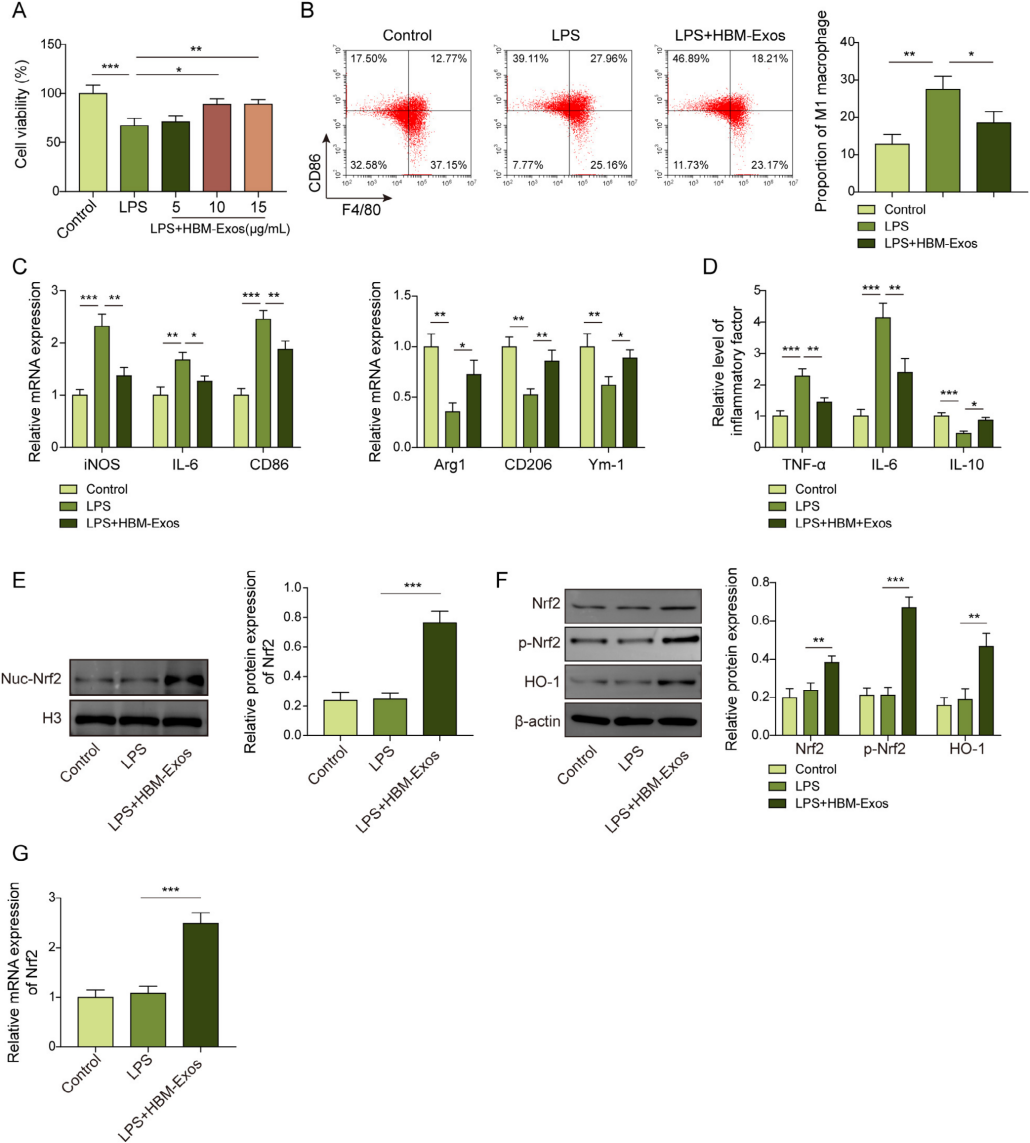
HBM‐Exos inhibited M1 macrophage polarization and activated Nrf2/HO‐1 signaling in LPS‐stimulated Kupffer cells. Kupffer cells were stimulated with LPS (1 μg/mL) for 16 h and treated with HBM‐Exos (10 μg/mL). (A) Cell viability was evaluated employing the CCK‐8 assays. (B) Flow cytometry was used to detect M1 macrophage markers CD86 and F4/80. (C) RT‐qPCR analysis was used to measure iNOS, IL‐6, CD86, Arg1, CD206, Ym‐1 cytokine levels. (D) Levels of TNF‐α, IL‐6, and IL‐10 inflammatory cytokines were evaluated using ELISA. (E) Western blotting was performed to determine the protein levels of Nrf2 in the nucleus. (F) Western blotting was performed to determine the protein levels of Nrf2, p‐Nrf2, and HO‐1. (G) RT‐qPCR analysis was performed to measure Nrf2 levels. Each detection was performed in triplicate. Data are presented as means ± SD, *n* = 3 per group. *p < 0.05; **p < 0.01; ***p < 0.001.

In HBM‐Exo‐treated Kupffer cells under LPS stimulation, the expression levels of M1‐associated cytokines (iNOS, IL‐6, and CD86) were significantly decreased, while M2‐associated cytokines (Arg1, CD206, and Ym‐1) were upregulated, thus demonstrating a clear dose‐dependent response to HBM‐Exos (5, 10, 15, and 20 μg/mL) (Figure S3A). Moreover, the LPS‐induced increase in inflammatory cytokine levels (TNF‐α and IL‐6) and decrease in IL‐10 levels in Kupffer cells were also alleviated by HBM‐Exo in a dose‐dependent manner (Figure S3B). Similarly, the expression of Nrf2 and HO‐1 pathway proteins showed a dose‐dependent activation, with more pronounced and stable experimental effects observed when HBM‐Exos were administered at 10 μg/mL (Figure S3C). As HBM‐Exo concentration increased, the expression level of FP671120.4 in LPS‐treated Kupffer cells also gradually rose, supporting the selection of 10 μg/mL as the optimal treatment concentration (Figure S3D). As shown in Figure 2A, HBM‐Exos treatment significantly up‐regulated the cell viabilities of LPS‐treated Kupffer cells, in a dosage manner (5, 10, and 15 μg/mL), and we selected the 10 μg/mL as the formal HBM‐Exos treatment concentration. LPS markedly increased macrophage M1 polarization (both CD86 and F4/80 positive) in Kupffer cells, whereas HBM‐Exos treatment reversed this trend (Figure 2B). Moreover, LPS promoted M1 macrophage cytokine (iNOS, IL‐6, and CD86) gene expression, while repressing the expression of M2 macrophage cytokines (Arg1, CD206, and Ym‐1) in Kupffer cells. In contrast, co‐treatment with HBM‐Exos overturned the effects of LPS, which downregulated M1 cytokine expression and upregulated M2 cytokines (Figure 2C). Similarly, HBM‐Exos treatment reversed the LPS‐induced increase in TNF‐α and IL‐6 levels in Kupffer cells, while reversing the downregulation of IL‐10 (Figure 2D). Nrf2 nuclear translocation always represents an increase in antioxidant‐related cellular protective mechanisms [24]. Compared to LPS treatment alone, co‐treatment with HBM‐Exos significantly enhanced the nuclear translocation of Nrf2 and increased the expression of Nrf2, p‐Nrf2, and HO‐1 in Kupffer cells (Figure 2E,F). Furthermore, HBM‐Exos also upregulated Nrf2 mRNA expression levels in Kupffer cells (Figure 2G). Collectively, these findings demonstrate that HBM‐Exos inhibit macrophage M1 polarization and activate the Nrf2/HO‐1 signaling pathway in LPS‐induced Kupffer cells.

### 3.3 HBM‐Exos Inhibited Macrophage M1 Polarization by Activating Nrf2/HO‐1 Signaling in LPS‐Induced Kupffer Cells

**FIGURE 3:**
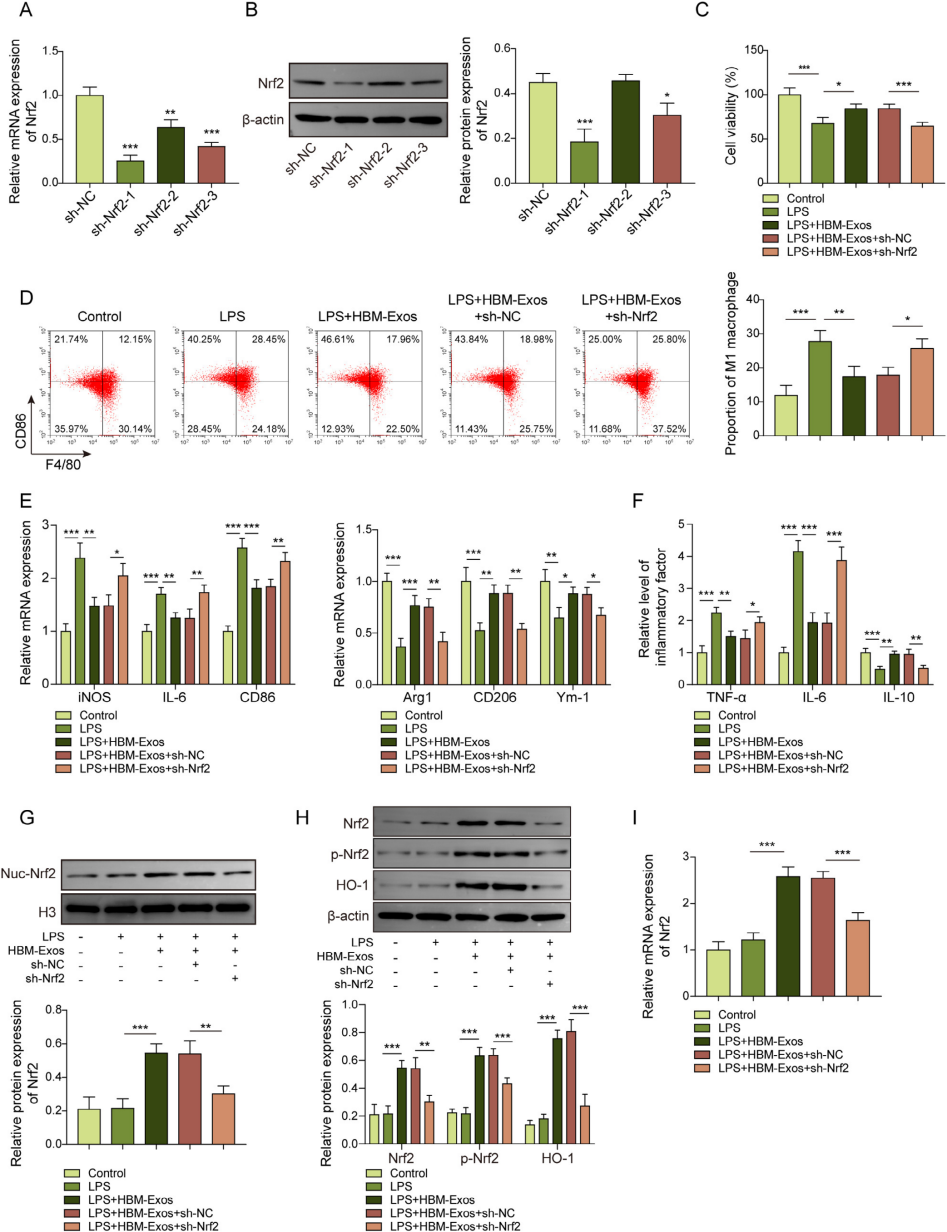
HBM‐Exos inhibited M1 macrophage polarization by upregulating Nrf2 expression via activation of Nrf2/HO‐1 signaling in LPS‐induced Kupffer cells. Kupffer cells were stimulated with LPS (1 μg/mL) for 16 h, treated with HBM‐Exos (10 μg/mL), and then infected with sh‐NC or sh‐Nrf2. (A) RT‐qPCR analysis was performed to measure Nrf2 levels. (B) Western blot was performed to measure Nrf2 protein levels. (C) Cell viability was evaluated using a CCK‐8 assay. (D) Flow cytometry was used to detect the M1 macrophage markers CD86 and F4/80. (E) RT‐qPCR analysis was performed to measure the levels of iNOS, IL‐6, CD86, Arg1, CD206, and Ym‐1 cytokines. (F) Levels of TNF‐α, IL‐6, and IL‐10 inflammatory cytokines were evaluated using ELISA. (G) Western blotting was used to determine the protein levels of Nrf2 in the cell nucleus (H) and to assess the protein levels of Nrf2, p‐Nrf2, and HO‐1. (I) RT‐qPCR analysis was performed to measure Nrf2 levels. Each measurement was performed in triplicate. Data are presented as means ± SD, *n* = 3 per group. *p < 0.05; **p < 0.01; ***p < 0.001.

To further investigate Nrf2's role and mechanism in HBM‐Exos‐mediated modulation of LPS‐induced macrophage M1 polarization, Kupffer cells were treated with LPS and HBM‐Exos and infected with sh‐Nrf2 or sh‐NC lentivirus (Nrf2 knockdown model). As shown in (Figure 3A,B), the gene and protein expression of Nrf2 was significantly decreased in the sh‐Nrf2‐1 group compared to that in the sh‐NC group, suggesting the successful establishment of the Nrf2 silencing model. Furthermore, HBM‐Exos recovered cell viability, which was abolished by Nrf2 silencing in LPS‐induced Kupffer cells (Figure 3C). Similarly, the HBM‐Exos‐mediated decrease in expression of macrophage M1 polarization markers CD86 and F4/80 and M1 macrophage cytokines and the increase in M2 macrophage cytokines were reversed by Nrf2 silencing in LPS‐treated Kupffer cells (Figure 3D,E). The increase in inflammatory cytokines (TNF‐α and IL‐6) and the downregulation of anti‐inflammatory factors (IL‐10) induced by HBM‐Exos were also reversed by Nrf2 silencing in LPS‐stimulated Kupffer cells (Figure 3F). In addition, compared with the LPS + HBM‐Exos treatment group, Nrf2 silencing significantly reduced Nrf2 nuclear translocation as well as the protein levels of total Nrf2, p‐Nrf2, and HO‐1 (Figure 3G,H). Consistently, Nrf2 silencing also reversed the HBM‐Exos‐induced upregulation of Nrf2 mRNA levels in LPS‐stimulated Kupffer cells (Figure 3I). Overall, our data showed that HBM‐Exos inhibited macrophage M1 polarization by activating Nrf2/HO‐1 signaling in LPS‐induced Kupffer cells.

### 3.4 Identification and Enrichment Analysis of lncRNA in HBM‐Exos

**FIGURE 4:**
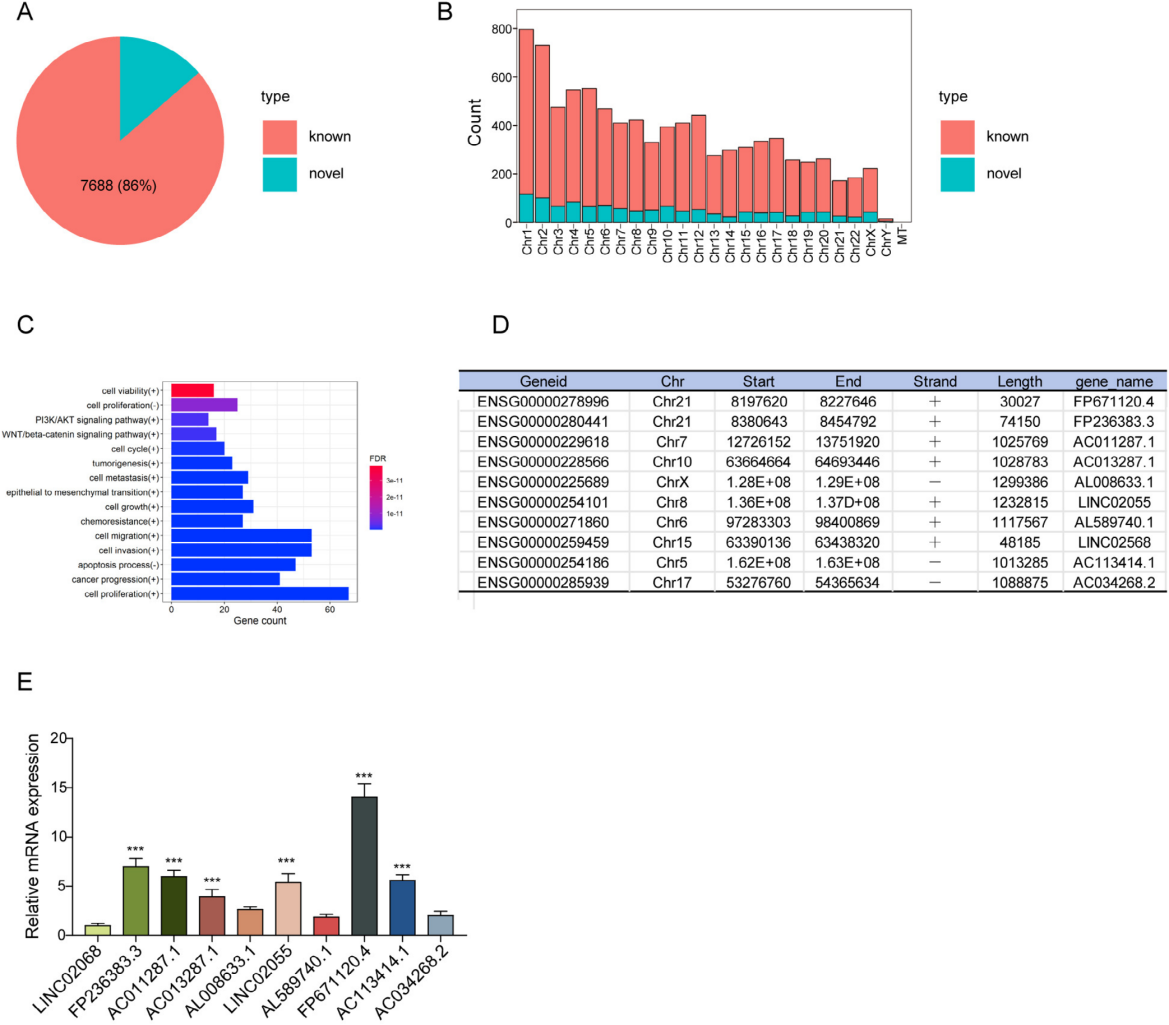
Identification and enrichment analysis of lncRNA in HBM‐Exos. (A) Known and novel proportions of identified lncRNAs. (B) Length distribution of identified lncRNAs. (C) LncRNAs functional enrichment analysis. (D) Top 10 enriched lncRNAs in HBM‐Exos. (E) RT‐qPCR analysis was used to validate the Top 10 enriched lncRNAs. Each detection was performed in triplicate. Data are presented as means ± SD, *n* = 3 per group. *p < 0.05; **p < 0.01; ***p < 0.001.

To further explore the regulatory mechanism of HBM‐Exos in LPS‐induced M1 polarization of Kupffer cells, the collected HBM‐Exos were subjected to lncRNA sequencing to analyze their expression profiles. As shown in Figure 4A,B, the identified lncRNAs were analyzed in proportion to known and novel groups, and their lengths were evaluated. Furthermore, lncRNAs with the top 10% expression levels were selected, and functional enrichment analysis was performed based on the LncTarD2.0 database (Figure 4C). Subsequently, the top 10 enriched lncRNAs of HBM‐Exos were listed and validated by RT‐qPCR analysis. The results identified FP671120.4 as the most differentially expressed lncRNA in HBM‐Exos (Figure 4D,E). This suggests that FP671120.4 may play a vital role in the HBM‐Exos‐mediated regulation of LPS‐induced M1 polarization in Kupffer cells.

### 3.5 FP671120.4 Silencing Reversed the HBM‐Exos‐Mediated Increase in Nrf2 mRNA Stability

**FIGURE 5:**
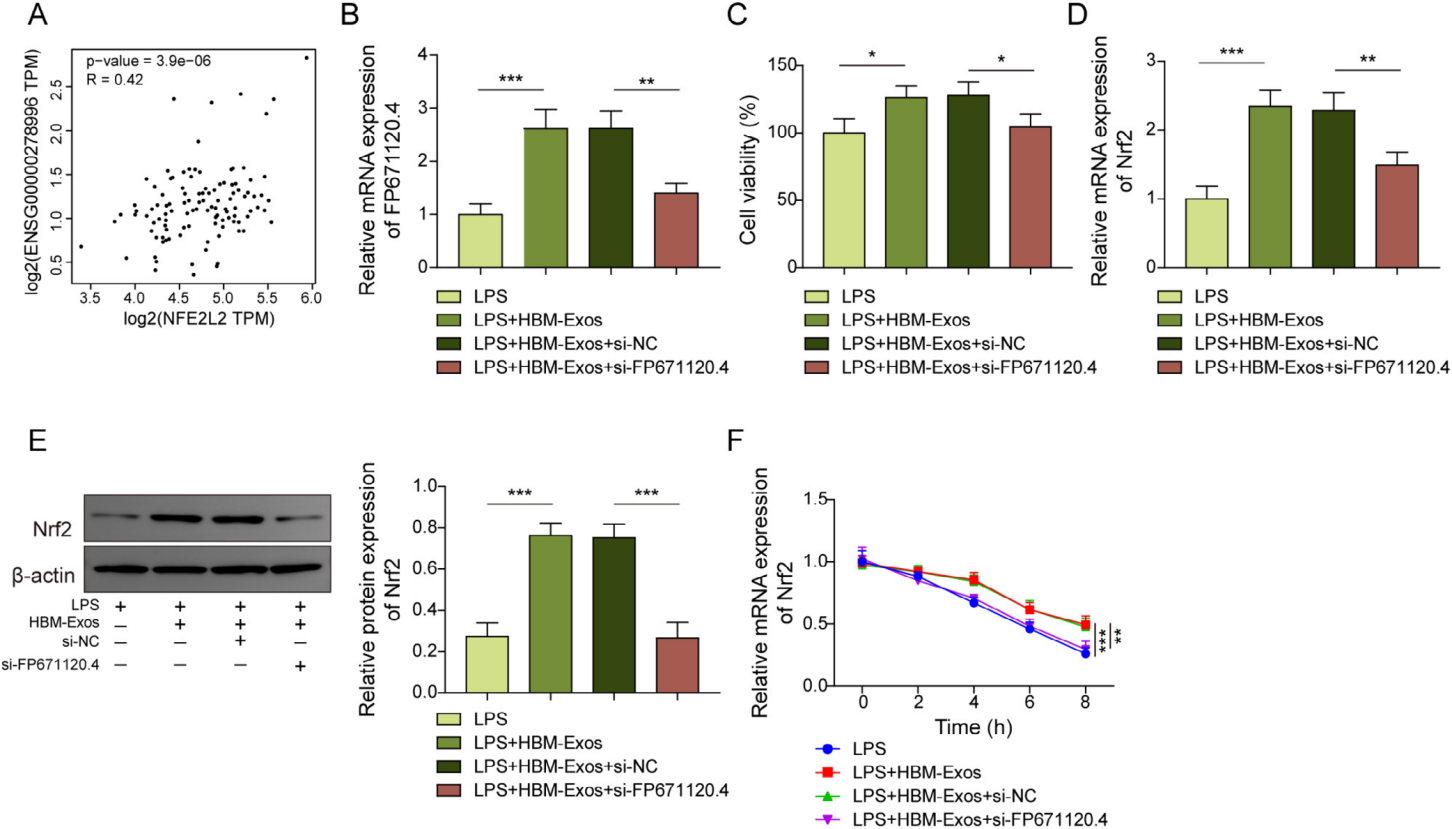
FP671120.4 silencing reversed the HBM‐Exos‐mediated increase in Nrf2 mRNA stability. (A) Correlation analysis between FP671120.4 and Nrf2 expression in the liver. Kupffer cells were stimulated with LPS (1 μg/mL) for 16 h, treated with HBM‐Exos (10 μg/mL), and infected with sh‐NC or sh‐FP671120.4. (B–F). (B) RT‐qPCR analysis was used to detect FP671120.4 levels. (C) Cell viability was evaluated using the CCK‐8 assay. (D) RT‐qPCR analysis was used to detect the Nrf2 levels. (E) Western blot assays were used to determine the protein level of Nrf2. (F) Nrf2 RNA decay assay. Each detection was performed in triplicate. Data are exhibited as means ± SD, *n* = 3 per group. *p < 0.05; **p < 0.01; ***p < 0.001.

As shown in Figure 5A, FP671120.4 expression was positively correlated with Nrf2 expression in liver tissue. Furthermore, to clarify the potential mechanism of HBM‐Exos‐derived FP671120.4 in LPS‐stimulated M1 polarization, FP671120.4 was silenced in LPS‐stimulated Kupffer cells treated with HBM‐Exos. HBM‐Exos treatment significantly enhanced FP671120.4 expression in LPS‐treated Kupffer cells, while this effect was reversed following si‐FP671120.4 transfection (Figure 5B). Furthermore, HBM‐Exos upregulated the viability of LPS‐induced Kupffer cells, but this effect was attenuated upon FP671120.4 silencing (Figure 5C). In addition, the HBM‐Exos‐induced increase in Nrf2 mRNA and protein expression was abolished by FP671120.4 silencing (Figure 5D,E). HBM‐Exos enhanced the mRNA stability of Nrf2; however, additional silencing of FP671120.4 negated the effect of HBM‐Exos (Figure 5F). Therefore, we concluded that FP671120.4 silencing overturned the HBM‐Exos‐mediated increase in Nrf2 mRNA stability.

### 3.6 HBM‐Exos‐Derived FP671120.4 Enhances the Interaction Between ELAVL1 and Nrf2

**FIGURE 6:**
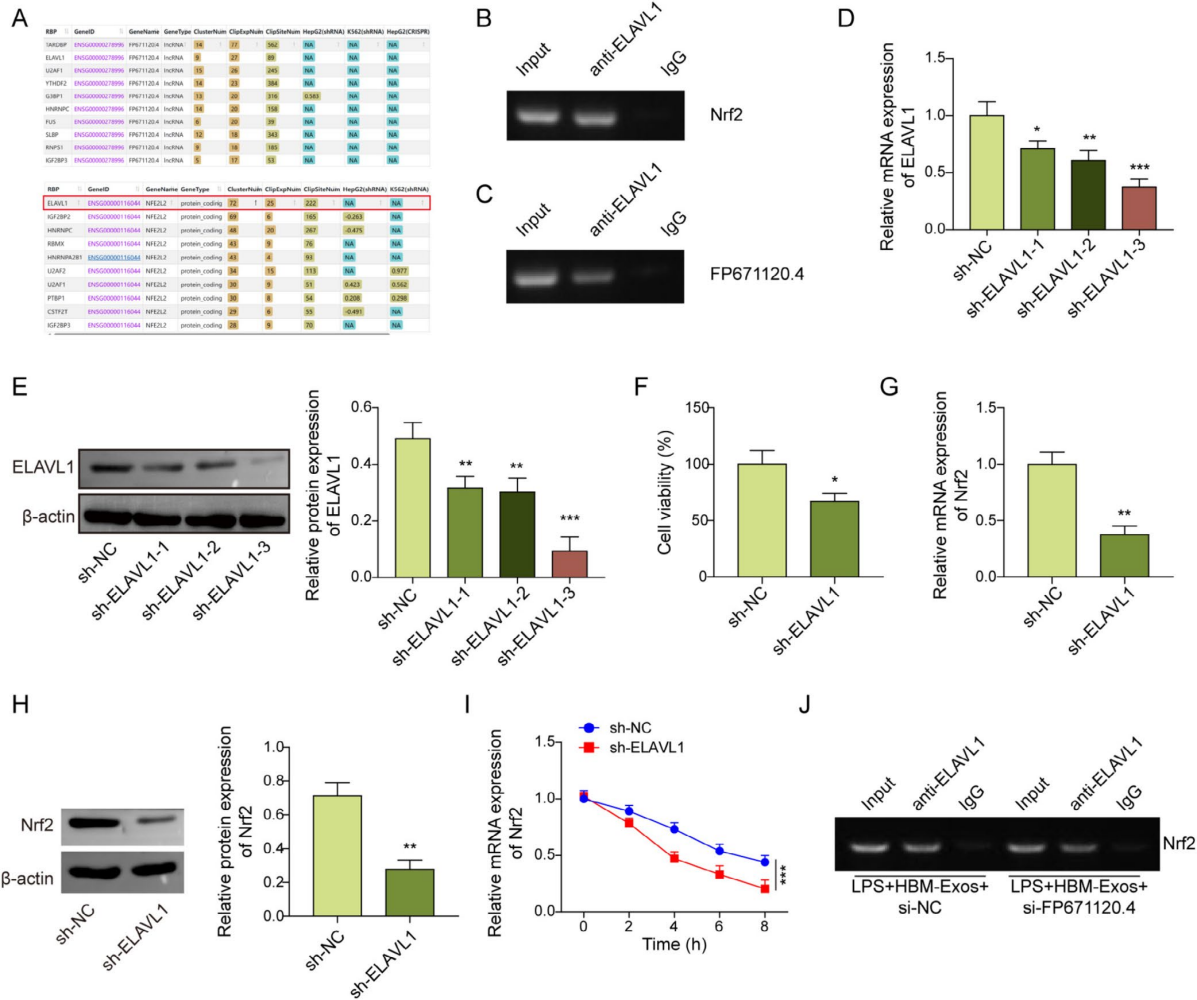
HBM‐Exos‐derived FP671120.4 enhanced the interaction between ELAVL1 and Nrf2. (A) The ENCORI database was used to predict the binding between FP671120.4 and ELAVL1 or Nrf2 and ELAVL1. Kupffer cells were stimulated with LPS (1 μg/mL) for 16 h, treated with HBM‐Exos (10 μg/mL), and infected with sh‐NC or sh‐ELAVL1. (B–I). (B) The interaction between Nrf2 and ELAVL1 was validated using RIP analysis. (C) The interaction between FP671120.4 and ELAVL1 was validated using RIP analysis. (D) RT‐qPCR analysis was employed to detect ELAVL1 levels. (E) Western blot assays were used to determine the protein levels of ELAVL1. (F) Cell viability was evaluated using the CCK‐8 assay. (G) RT‐qPCR analysis was used to detect Nrf2 levels. (H) Western blot assays were used to determine Nrf2 protein levels. (I) Nrf2 RNA decay assay. Kupffer cells were stimulated with LPS (1 μg/mL) for 16 h, treated with HBM‐Exos (10 μg/mL) and infected with sh‐NC or sh‐FP671120.4. (J) The interaction between Nrf2 and ELAVL1 was confirmed using RIP analysis. Each detection was performed in triplicate. Data are presented as means ± SD, *n* = 3 per group. *p < 0.05; **p < 0.01; ***p < 0.001.

As shown in Figure 6A, potential binding interactions between FP671120.4 and ELAVL1, as well as between Nrf2 and ELAVL1, were identified using the ENCORI database (http://starbase.sysu.edu.cn/index.php). Consistently, RIP analysis confirmed the interaction between FP671120.4, ELAVL1, Nrf2, and ELAVL1 (Figure 6B,C). Subsequently, Kupffer cells were transduced with sh‐ELAVL1 or sh‐NC, resulting in the downregulation of ELAVL1 mRNA and protein expression (Figure 6D,E). ELAVL1 silencing significantly reduced the viability of Kupffer cells compared to the sh‐NC group (Figure 6F). Moreover, ELAVL1 knockdown markedly decreased the mRNA and protein levels of Nrf2 in Kupffer cells (Figure 6G,H), as well as the mRNA stability of Nrf2 (Figure 6I). Following LPS + HBM‐Exos treatment, FP671120.4 silencing attenuated the interaction between ELAVL1 and Nrf2 (Figure 6J). Together, these results confirm the interactions among FP671120.4, ELAVL1, Nrf2, and ELAVL1, and demonstrate that HBM‐Exos‐derived FP671120.4 could enhance the interaction between Nrf2 and ELAVL1 in LPS‐stimulated Kupffer cells.

### 3.7 HBM‐Exos‐Derived FP671120.4 Inhibits Macrophage M1 Polarization by Promoting Nrf2 Expression via Activation of the Nrf2/HO‐1 Pathway in LPS‐Stimulated Kupffer Cells

**FIGURE 7:**
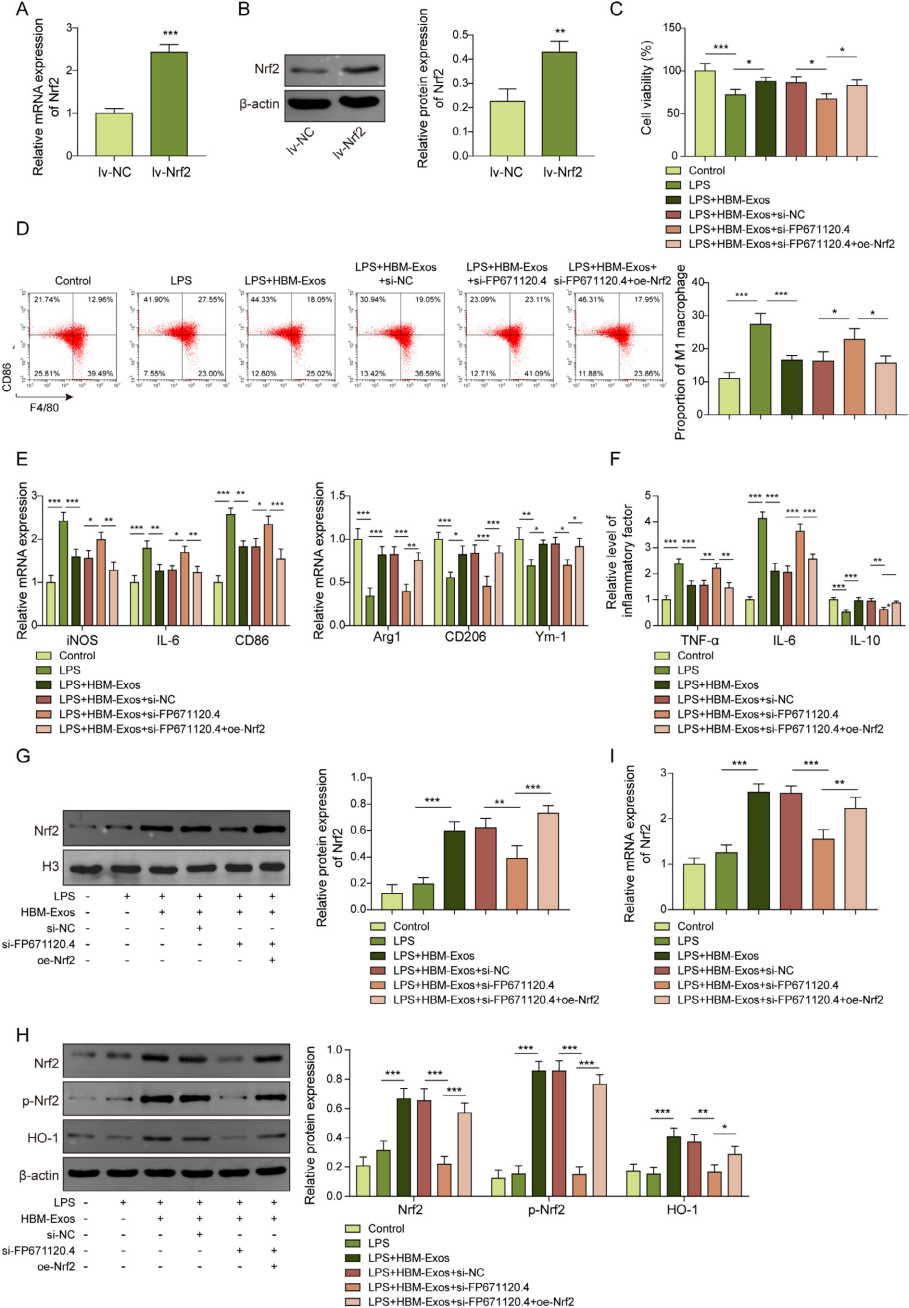
HBM‐Exos‐derived FP671120.4 inhibited macrophage M1 polarization by promoting Nrf2 expression via activation of the Nrf2/HO‐1 pathway in LPS‐stimulated Kupffer cells. Kupffer cells were stimulated with LPS (1 μg/mL) for 16 h, treated with HBM‐Exos (10 μg/mL) and infected with sh‐NC or sh‐FP671120.4 and oe‐NC or oe‐Nrf2. (A) RT‐qPCR analysis was performed to detect Nrf2 levels. (B) Western blot was performed to determine Nrf2 protein levels. (C) Cell viability was evaluated using a CCK‐8 assay. (D) Flow cytometry was used to detect the macrophage M1 markers CD86 and F4/80. (E) RT‐qPCR analysis was performed to detect iNOS, IL‐6, CD86, Arg1, CD206, and Ym‐1 cytokine levels. (F) Inflammatory cytokine levels of TNF‐α, IL‐6, and IL‐10 were evaluated using ELISA assays. (G) Western blotting was used to determine the protein levels of Nrf2 in the cell nucleus (H) and of Nrf2, p‐Nrf2, and HO‐1. (I) RT‐qPCR analysis was performed to detect Nrf2 levels. Each detection was performed in triplicate. Data are presented as means ± SD, *n* = 3 per group. *p < 0.05; **p < 0.01; ***p < 0.001.

As shown in Figure 7A,B, overexpression of Nrf2 (oe‐Nrf2) significantly increased Nrf2 expression at both mRNA and protein levels compared to the negative control (oe‐NC). Kupffer cells were then treated with HBM‐Exos, FP671120.4 silencing, and Nrf2 overexpression under LPS stimulation. Silencing FP671120.4 reversed the HBM‐Exos‐induced enhancement of cell viability in LPS‐stimulated Kupffer cells, while co‐treatment with Nrf2 overexpression counteracted the effects of FP671120.4 knockdown (Figure 7C). In the presence of LPS, FP671120.4 silencing abolished the inhibitory effect of HBM‐Exos on M1 polarization. However, Nrf2 overexpression markedly mitigated the negative effects of FP671120.4 silencing (Figure 7D–F). In addition, HBM‐Exos‐induced nuclear accumulation of Nrf2, increased Nrf2 phosphorylation, and activation of the Nrf2/HO‐1 signaling pathway were suppressed by FP671120.4 silencing. These inhibitory effects were reversed by co‐treatment with Nrf2 overexpression (Figure 7G,H). Consistently, the HBM‐Exo‐mediated increase in Nrf2 mRNA levels was reversed by FP671120.4 silencing, whereas Nrf2 co‐overexpression restored Nrf2 expression in LPS‐stimulated Kupffer cells (Figure 7I). Collectively, our experimental data indicate that HBM‐Exos‐derived FP671120.4 could inhibit macrophage M1 polarization by enhancing Nrf2 expression through the activation of the Nrf2/HO‐1 pathway in LPS‐stimulated Kupffer cells.

## 4 Discussion

Sepsis is widely recognized as a serious medical condition with extremely high mortality and morbidity rates (affecting approximately 30 million patients worldwide per year). It is also associated with a significant burden on the healthcare system [25]. The uncontrolled and excessive inflammatory response characteristic of sepsis can lead to widespread organ dysfunction, injury, and even failure [26]. The liver plays a central role in host immune defense, and sepsis‐associated liver injury (SALI) is a major complication that adversely affects both mortality and prognosis in patients with sepsis [27]. However, targeted and feasible therapies to control the occurrence and development of SALI are lacking. Kupffer cells, the liver's resident macrophages, are critical regulators of SALI progression through their polarization states [28]. HBM‐Exos are positive regulators of inflammation and infections. However, the precise role and mechanism by which HBM‐Exos modulate macrophage polarization in Kupffer cells during SALI remain unclear. In this study, we demonstrated that HBM‐Exos‐derived FP671120.4 can repress M1 polarization by modulating the ELAVL1/Nrf2/HO‐1 pathway in LPS‐induced Kupffer cells, a SALI cell model. These findings suggest that HBM‐Exos may serve as a promising therapeutic agent for SALI, highlighting the ELAVL1/Nrf2/HO‐1 axis as a potential diagnostic or therapeutic target for this condition.

HBM is an essential source of nutrition during infancy and early life, playing a significant role in supporting immune development and function. Studies have shown that HBM positively influences the development of the host immune system and mitigates excessive inflammatory reactions associated with various diseases in infants [12, 29]. As such, HBM holds potential for protecting newborns from sepsis and other inflammatory diseases. This immunomodulatory capacity is partly attributed to bioactive vehicles, such as exosomes. In this study, we successfully isolated exosomes from HBM, and their morphological characteristics and expression of exosomal markers were consistent with previous findings [30]. We further confirmed that HBM‐Exos were internalized by Kupffer cells during co‐treatment, aligning with earlier observations [17]. Furthermore, we found that HBM‐Exos significantly inhibited LPS‐induced M1 macrophage polarization and suppressed the excessive release of inflammatory cytokines in Kupffer cells, an established in vitro model for SALI. The observed anti‐inflammatory effect of HBM‐Exos parallels the previously reported suppressive effects of HBM in LPS‐treated organoids within a necrotizing enterocolitis model [31]. Notably, our study is the first to validate that HBM‐Exos can inhibit M1 polarization in LPS‐induced Kupffer cells, thus extending the known anti‐inflammatory mechanisms of HBM. Moreover, the activation of Nrf2/HO‐1 signaling has been shown to suppress M1 polarization and help regulate SALI [9]. Under oxidative stress, Nrf2 undergoes various post‐translational modifications, such as phosphorylation [32]. Specifically, phosphorylation at Ser40, mediated by protein kinase C (PKC), facilitates Nrf2's translocation to the nucleus, enabling a protective response to oxidative damage [33]. We also observed that HBM‐Exos enhanced Nrf2 expression, promoted its nuclear translocation, increased p‐Nrf2 levels, and activated Nrf2/HO‐1 signaling in LPS‐treated Kupffer cells. This aligns with previous evidence indicating that HBM can downregulate inflammation by upregulating Nrf2 protein expression [34]. Therefore, our findings demonstrate that HBM‐Exos suppress M1 polarization in LPS‐stimulated Kupffer cells, highlighting their potential function in the management of SALI.

HBM‐Exos exert immunomodulatory effects during newborn development through exosome‐associated signaling molecules, including lncRNAs. For instance, HBM‐exosomal lncRNAs have been shown to potentially protect against necrotizing enterocolitis [19]. However, to date, no published studies have specifically investigated the role of HBM‐exosomal lncRNAs in macrophage polarization during SALI. In this study, we performed RNA sequencing on isolated HBM‐Exos and identified FP671120.4 as the most highly expressed lncRNA. Currently, research on the biological function of FP671120.4 is limited; however, our findings suggest it may play a regulatory role in the HBM‐Exos‐mediated suppression of M1 polarization in LPS‐induced Kupffer cells. Notably, we report for the first time that FP671120.4 expression is positively correlated with Nrf2 expression in the liver. Furthermore, silencing FP671120.4 reversed the HBM‐Exos‐mediated increase in cell viability in LPS‐treated Kupffer cells. We also demonstrated that FP671120.4 positively regulates Nrf2 expression, enhances Nrf2 phosphorylation, and upregulates Nrf2 mRNA stability in Kupffer cells. Based on the inhibitory function of Nrf2 in M1 polarization, FP671120 inhibits M1 polarization by upregulating Nrf2 expression in LPS‐treated Kupffer cells [35]. This study provides new insights into the specific function and mechanism of HBM‐Exosomal FP671120.4 in regulating macrophage polarization and expands our understanding of how HBM‐Exos contribute to the modulation of immune responses in the context of SALI. Nrf2, as a master regulator of the antioxidant response, is subject to complex regulation at multiple levels, including transcriptional initiation, chromatin remodeling, and post‐translational modifications (PTMs) such as phosphorylation and ubiquitination. Moreover, lncRNAs can regulate the expression and function of Nrf2 through various modulatory mechanisms. For instance, lncRNA SNAI3‐AS1 could bind to staphylococcal nuclease domain‐containing protein 1 (SND1) and disrupt the m6A‐dependent recognition of Nrf2 mRNA 3′‐UTR by SND1, thereby downregulating the mRNA stability of Nrf2 [36]. In addition, lncRNA TUG1 could also modulate Nrf2 expression by regulating miR‐144‐3p in renal tubular epithelial cells [37]. LncRNA LAMTOR5‐AS1 could obstruct the ubiquitin‐mediated degradation pathway of Nrf2, leading to an increased level of Nrf2, while also diminishing its transcriptional activity [38]. In future studies, we plan to conduct in‐depth investigations into the potential role of FP671120.4 in the transcriptional regulation of Nrf2. We aim to explore possible effects on post‐transcriptional modifications of Nrf2, including but not limited to phosphorylation, ubiquitination, and acetylation. These studies will help us systematically elucidate the regulatory network between FP671120.4 and Nrf2, thereby providing a more comprehensive understanding of their functional relationship.

ELAVL1 is a member of the RNA‐binding protein family and is known to stabilize target mRNAs by binding to their 3′‐UTR regions. LncRNAs often regulate downstream target genes by recruiting specific RNA‐binding proteins to modulate biological processes. For instance, lncRNA GMDS‐AS1 has been shown to stabilize SIRT1 mRNA by recruiting TAF15, thereby influencing the progression of lung adenocarcinoma [39]. According to bioinformatics predictions, FP671120.4 may potentially bind to ELAVL1. In this study, we confirmed that FP671120.4 directly interacts with ELAVL1, and that ELAVL1 binds to Nrf2 in Kupffer cells. Moreover, silencing ELAVL1 resulted in reduced Nrf2 expression and decreased Nrf2 mRNA stability. This finding aligns with previous reports demonstrating a positive interaction between ELAVL1 and Nrf2 in a retinal pigment epithelium model [40]. Importantly, silencing FP671120.4 significantly disrupted the interaction between ELAVL1 and Nrf2 in Kupffer cells, which is a novel finding. Together, these results elucidate a positive regulatory interaction among FP671120.4, ELAVL1, and Nrf2 in Kupffer cells. Moreover, we demonstrated that HBM‐Exossomal FP671120.4 inhibits M1 polarization by activating the Nrf2/HO‐1 signaling pathway through upregulation of Nrf2. These findings suggest that the FP671120.4/ELAVL1/Nrf2 axis plays a critical role in the HBM‐Exos‐mediated regulation of M1 polarization in LPS‐stimulated Kupffer cells.

In summary, this study demonstrated that HBM‐derived exosomal FP671120.4 inhibits M1 polarization by regulating the ELAVL1/Nrf2 axis in an LPS‐induced cellular model of SALI. Further validation using a sepsis mouse model is necessary to confirm the molecular mechanisms identified and to extend their translational relevance. However, due to current experimental limitations, we were unable to conduct in vivo validation. Subject to future feasibility, we plan to perform animal experiments using established sepsis models, such as the cecal ligation and puncture model. The mechanisms uncovered in this study require in vivo confirmation, which will be an important focus of future investigations. Clarifying the specific function and mechanism of HBM‐Exos could enhance their potential application in SALI therapy. Moreover, the ELAVL1/Nrf2 signaling pathway may serve as a molecular target for the treatment of SALI.

## Ethics Statement

This study protocol was reviewed and approved by the Medical Ethics Committee of Affiliated Zhangzhou Hospital of Fujian Medical University [No. 2023kyz281].

## Conflicts of Interest

The authors declare no conflicts of interest.

## Supporting information


**Supplementary Figure 1.** Evaluation of the stability of HBM‐Exomal FP671120.4 derived from different donors. (A) RT‐qPCR analysis was employed to detect the FP671120.4 level, and the coefficient of variation among donors was analyzed. (B) Western blot assays were utilized to determine the protein density of CD63 and TSG101. Each detection was performed in triplicates. Data was exhibited as means ± SD, *n* = 20. ** *p* < 0.01; *** *p* < 0.001.

**Supplementary Figure 2.** Determination of the stability of HBM‐Exomal FP671120.4 under different storage conditions. HBM‐Exos were stored at −80°C, 4°C, and room temperature, as well as at different time points (0, 24, 72 h, and 1 week). (A) Agarose gel electrophoresis analysis was used to evaluate the RNA integrity of FP671120.4. (B) RT‐qPCR analysis was employed to detect the FP671120.4 level. (C) A transmission electron microscope was employed to observe the exosomal morphologies. Each detection was performed in triplicates. Data was exhibited as means ± SD, *n* = 3. ** *p* < 0.01; *** *p* < 0.001.

**Supplementary Figure 3.** Detection of the FP671120.4 expression and inflammatory function of the different concentrations of HBM‐Exos‐treated Kupffer cells with LPS stimulation. Kupffer cells were stimulated with LPS (1 μg/mL) for 16 h, and treated with HBM‐Exos (5, 10, and 15 μg/mL). (A) RT‐qPCR analysis was employed to detect iNOS, IL‐6, CD86, Arg1, CD206, and Ym‐1 cytokine levels. (B) Inflammatory cytokine levels of TNF‐α, IL‐6, and IL‐10 were evaluated using ELISA assays. (C) Western blot assays were utilized to determine the protein densities of Nrf2, p‐Nrf2, and HO‐1. Kupffer cells were stimulated with LPS (1 μg/mL) for 16 h, and treated with HBM‐Exos (1, 5, 10, 15, and 20 μg/mL). (D) RT‐qPCR analysis was employed to detect the FP671120.4 level. Each detection was performed in triplicates. Data was exhibited as means ± SD, *n* = 3. ** *p* < 0.01; *** *p* < 0.001.

**Table S1:** The primers for quantitative real‐time polymerase chain reaction assay.

## Data Availability

Data sharing not applicable to this article as no datasets were generated or analysed during the current study.
