# Supplementary figures and images for: Human Breast Milk‐Derived Exosomal FP671120.4 Inhibits Macrophage M1 Polarization via Modulating the ELAVL1/Nrf2 Axis in Sepsis‐Associated Liver Injury
Source: Kaohsiung J Med Sci. 2025 Sep 27;42(2):e70108. doi: 10.1002/kjm2.70108 (PMC12884777; doi:10.1002/kjm2.70108)

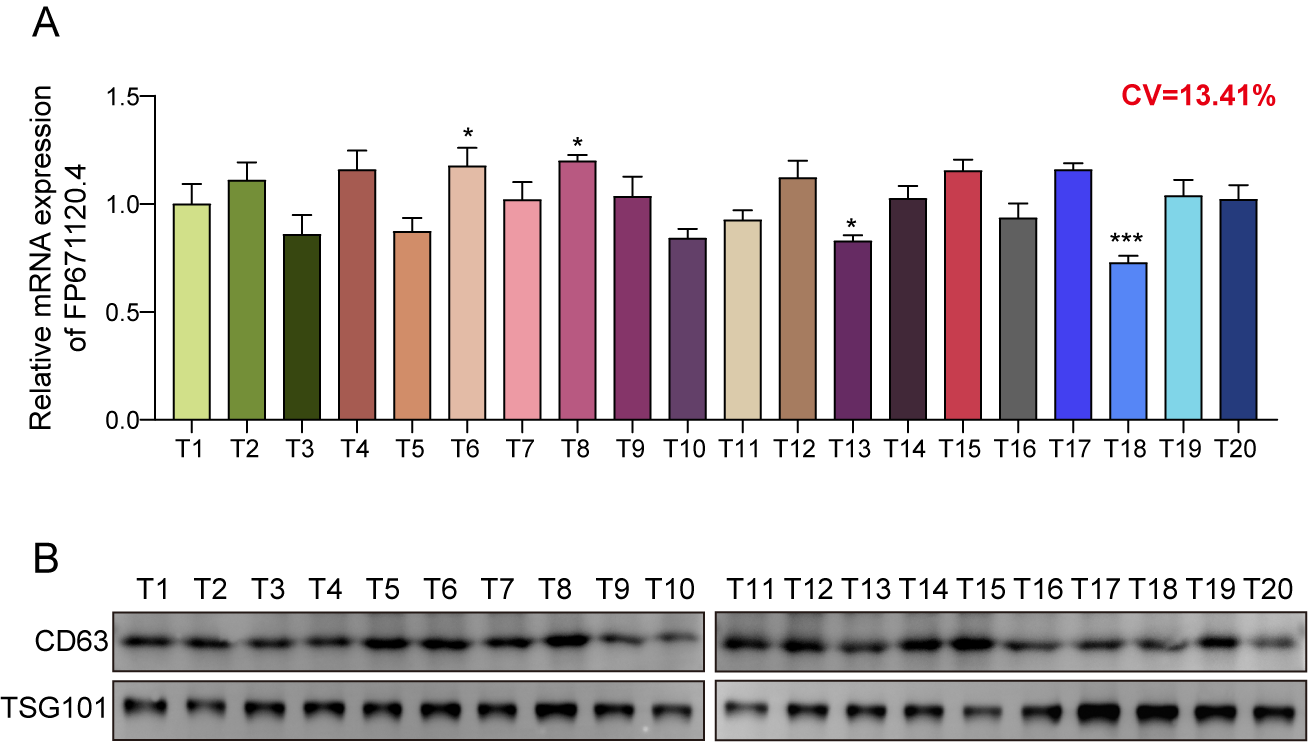

Supplement: Supplementary file 1 — Supplementary Figure 1. Evaluation of the stability of HBM‐Exomal FP671120.4 derived from different donors. (A) RT‐qPCR analysis was employed to detect the FP671120.4 level, and the coefficient of variation among donors was analyzed. (B) Western blot assays were utilized to determine the protein density of CD63 and TSG101. Each detection was performed in triplicates. Data was exhibited as means ± SD, n = 20. ** p < 0.01; *** p < 0.001. [file KJM2-42-e70108-s004.tif]

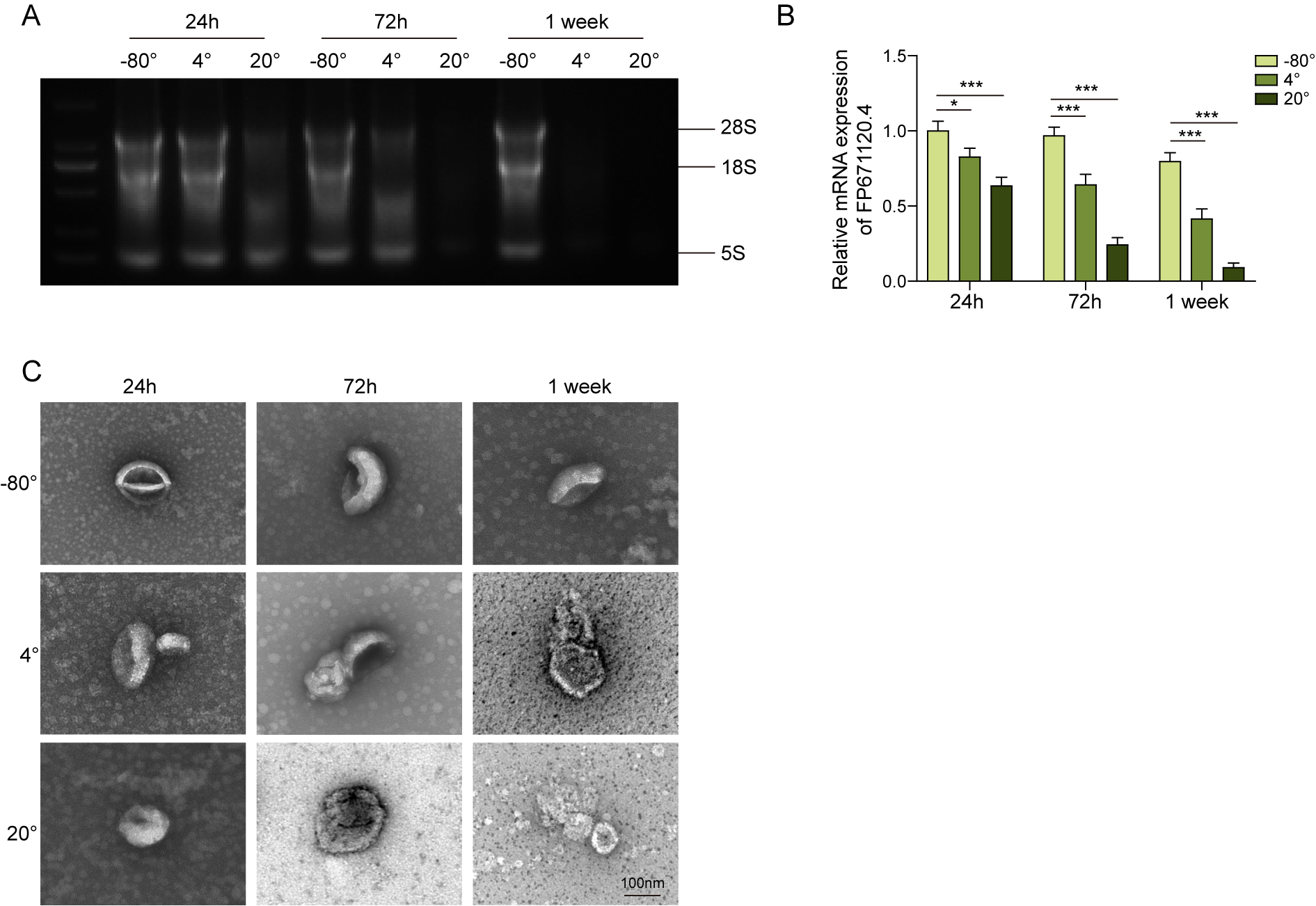

Supplement: Supplementary file 2 — Supplementary Figure 2. Determination of the stability of HBM‐Exomal FP671120.4 under different storage conditions. HBM‐Exos were stored at −80°C, 4°C, and room temperature, as well as at different time points (0, 24, 72 h, and 1 week). (A) Agarose gel electrophoresis analysis was used to evaluate the RNA integrity of FP671120.4. (B) RT‐qPCR analysis was employed to detect the FP671120.4 level. (C) A transmission electron microscope was employed to observe the exosomal morphologies. Each detection was performed in triplicates. Data was exhibited as means ± SD, n = 3. ** p < 0.01; *** p < 0.001. [file KJM2-42-e70108-s003.tif]

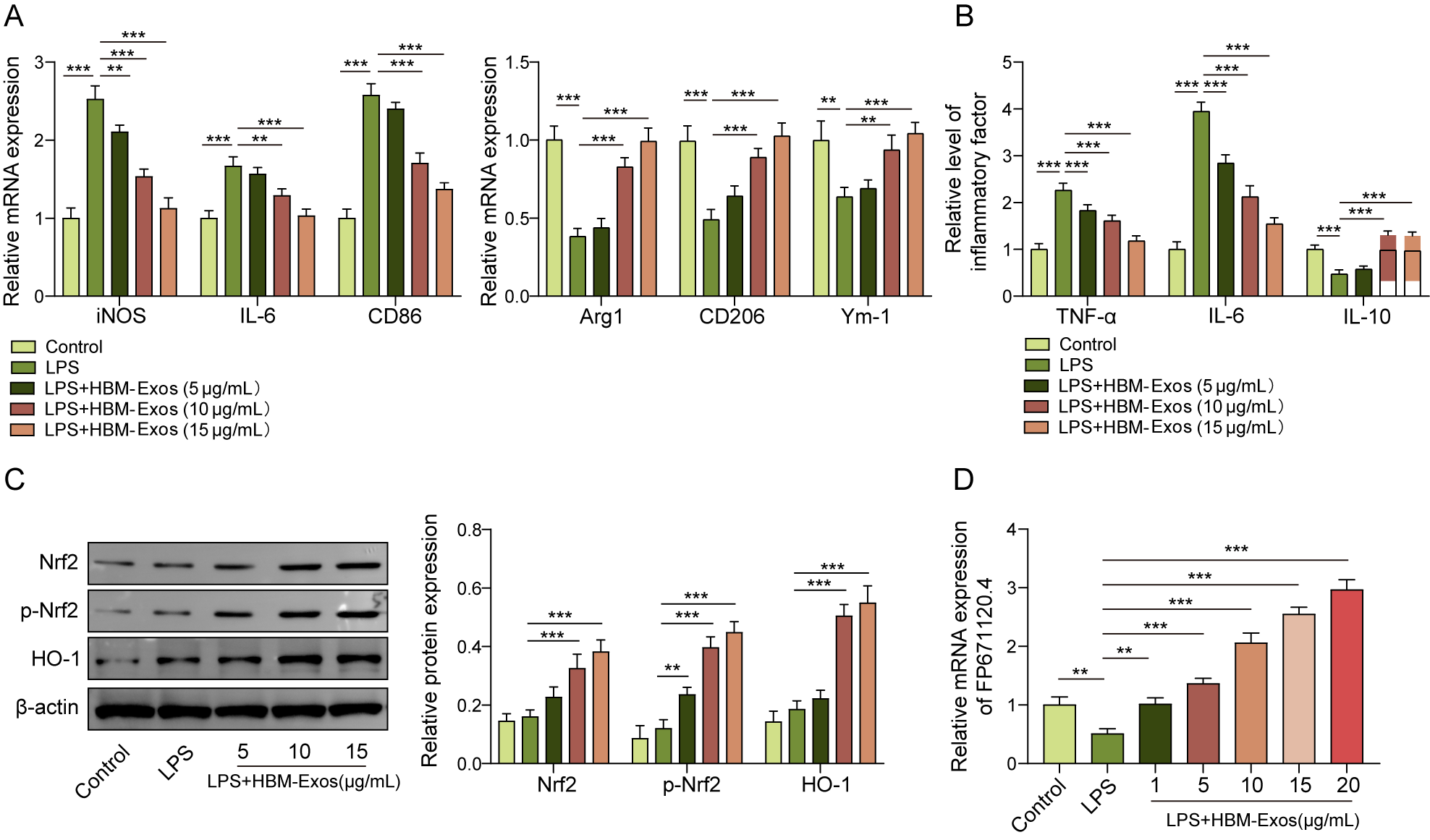

Supplement: Supplementary file 3 — Supplementary Figure 3. Detection of the FP671120.4 expression and inflammatory function of the different concentrations of HBM‐Exos‐treated Kupffer cells with LPS stimulation. Kupffer cells were stimulated with LPS (1 μg/mL) for 16 h, and treated with HBM‐Exos (5, 10, and 15 μg/mL). (A) RT‐qPCR analysis was employed to detect iNOS, IL‐6, CD86, Arg1, CD206, and Ym‐1 cytokine levels. (B) Inflammatory cytokine levels of TNF‐α, IL‐6, and IL‐10 were evaluated using ELISA assays. (C) Western blot assays were utilized to determine the protein densities of Nrf2, p‐Nrf2, and HO‐1. Kupffer cells were stimulated with LPS (1 μg/mL) for 16 h, and treated with HBM‐Exos (1, 5, 10, 15, and 20 μg/mL). (D) RT‐qPCR analysis was employed to detect the FP671120.4 level. Each detection was performed in triplicates. Data was exhibited as means ± SD, n = 3. ** p < 0.01; *** p < 0.001. [file KJM2-42-e70108-s001.tif]
